# Characterization of the minimum domain required for targeting budding yeast myosin II to the site of cell division

**DOI:** 10.1186/1741-7007-4-19

**Published:** 2006-06-26

**Authors:** Ida MB Lister, Nicola J Tolliday, Rong Li

**Affiliations:** 1 Department of Cell Biology, Harvard Medical School, 240 Longwood Ave, Boston, MA, 02115, USA; 2 Present address: Department of Molecular Biology and Microbiology, Tufts University School of Medicine, 136 Harrison Avenue, Boston, MA, 02111, USA; 3 Present address: Broad Institute of Harvard and MIT, 7 Cambridge Center, Cambridge, MA 02142, USA; 4 Present address: Stowers Institute for Medical Research, 1000 E. 50th Street, Kansas City, MO 64110, USA

## Abstract

**Background:**

All eukaryotes with the exception of plants use an actomyosin ring to generate a constriction force at the site of cell division (cleavage furrow) during mitosis and meiosis. The structure and filament forming abilities located in the C-terminal or tail region of one of the main components, myosin II, are important for localising the molecule to the contractile ring (CR) during cytokinesis. However, it remains poorly understood how myosin II is recruited to the site of cell division and how this recruitment relates to myosin filament assembly. Significant conservation between species of the components involved in cytokinesis, including those of the CR, allows the use of easily genetically manipulated organisms, such as budding yeast (*Saccharomyces cerevisiae*), in the study of cytokinesis. Budding yeast has a single myosin II protein, named Myo1. Unlike most other class II myosins, the tail of Myo1 has an irregular coiled coil. In this report we use molecular genetics, biochemistry and live cell imaging to characterize the minimum localisation domain (MLD) of budding yeast Myo1.

**Results:**

We show that the MLD is a small region in the centre of the tail of Myo1 and that it is both necessary and sufficient for localisation of Myo1 to the yeast bud neck, the pre-determined site of cell division. Hydrodynamic measurements of the MLD, purified from bacteria or yeast, show that it is likely to exist as a trimer. We also examine the importance of a small region of low coiled coil forming probability within the MLD, which we call the hinge region. Removal of the hinge region prevents contraction of the CR. Using fluorescence recovery after photobleaching (FRAP), we show that GFP-tagged MLD is slightly more dynamic than the GFP-tagged full length molecule but less dynamic than the GFP-tagged Myo1 construct lacking the hinge region.

**Conclusion:**

Our results define the intrinsic determinant for the localization of budding yeast myosin II and show it to be an oligomer of tentatively 3 monomers. We suggest that this is the minimum oligomeric unit (rather than the traditional myosin II dimer) that would allow specific assembly to the site of cytokinesis in a manner similar to the full length molecule. The flexible hinge region also contributes to CR structural integrity and contractility.

## Background

Budding yeast has one class II myosin, Myo1 [1,2], with the classic myosin superfamily domain organisation consisting of a conserved motor domain, a neck domain of 2 IQ motifs that binds the light chains Mlc1 and Mlc2 [3-5] and a tail region with a predicted coiled coil, as seen for all class II myosins so far studied. While the motor domain is responsible for the actin binding and ATP hydrolysis that allows the myosin to move along actin filaments, the class II myosin tails are responsible for how the motor function is used: the tails determine dimerisation, filament formation and localisation [6,7].

There are two potential ways in which class II myosins may localise to the site of cell division: 1) by binding to other proteins, as for example in fission yeast, (*Schizosaccharomyces pombe) *where Mid1 is shown to temporally regulate the localisation of the major class II myosin, Myo2, [8]; 2) by filament formation, as shown in *D. discoideum *where filament formation is required for myosin II to localise to the cleavage furrow [9,10]. In non-muscle, smooth muscle and *D. discoideum *myosin II, it has also been shown that one or more small regions in the tail are responsible for filament formation [7,9,11]. Filament formation is thought to occur by the interaction of the charged residues (exposed by coiled coil formation) between two dimers. With the exception of fission yeast Myp2, which has a discontinuous coiled coil and forms a monomer [12], the class II myosins studied to date all form dimers. In *D. discoideum*, filament formation also depends on dephosphorylation of 3 serine residues [7,9]. It was also shown that dephosphorylation of Myo2 in fission yeast is necessary for localisation [8,13]. It should be noted that higher eukaryotic class II myosins are also regulated by phosphorylation of their light chains but this has not been demonstrated in *D. discoideum *or fission yeast [7,14].

**Figure 1:**
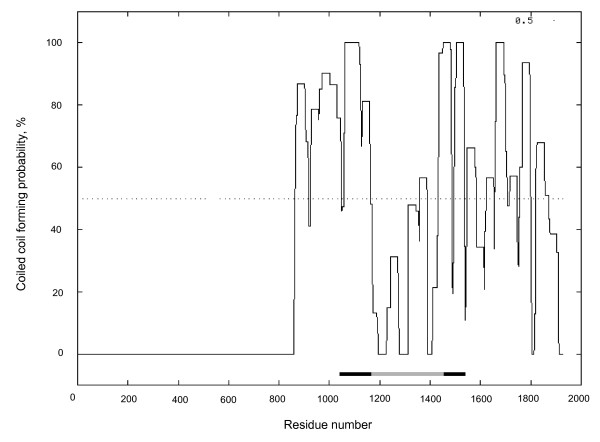
**Paircoil analysis of Myo1**. Coiled coil prediction of *S. Cerevisiae *Myo1 using the PAIRCOIL prediction program [15]. The black bar denotes the position of the MLD and the gray bar within the black bar denotes the position of the hinge region within the MLD. The dotted line is shown as a reference point for 50 % probability of coiled coil formation.

Very little is known about the structure of budding yeast Myo1 and how it is recruited to the site of cell division. Unlike higher eukaryotic class II myosins, Myo1 is not predicted to consist of a continuous coiled coil as shown in the PAIR COIL [15] prediction plot in Fig. 1. This raises the question as to what kind of structure the Myo1 tail forms and how this might affect its cellular function and the structure of the contractile ring (CR). In order to determine if coiled coil formation is required for the localization of budding yeast myosin II, we set out to map the minimum domain in the tail region that is necessary and sufficient for Myo1 localisation. We use hydrodynamic measurements to assess its oligomeric nature and use FRAP analysis to compare the *in vivo *dynamics of the minimum localisation domain with the full length molecule. We also demonstrate that a region of low coiled coil forming potential that we have named the hinge region is important in the localisation and function of Myo1.

## Results

### A small region in the tail is sufficient for Myo1 localisation

**Figure 2:**
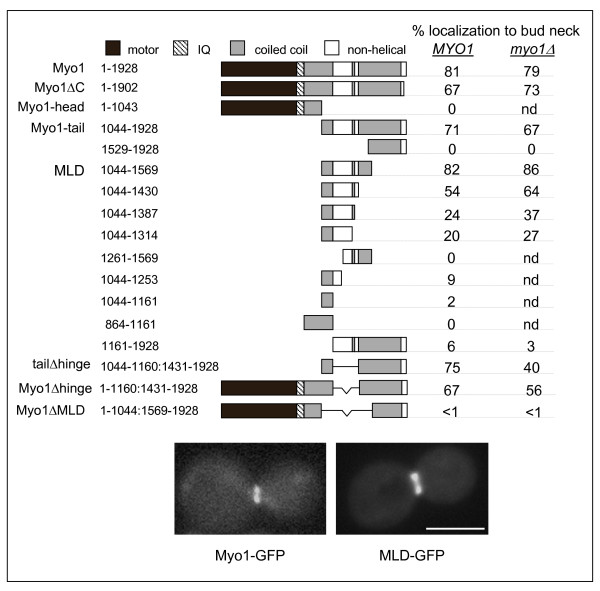
**Localisation of Myo1-GFP and deletion mutants in wild type and myo1 null yeast strains**. Budding yeast strains (*MYO1 *and *myo1Δ) *were transformed with Myo1 constructs tagged with GFP as shown here schematically. Fluorescence microscopy was used to quantify the number of fluorescent bud necks as a percentage of the total number of bud neck and these numbers are given on the right. Fluorescence microscopy images of budding yeast cells labeled with Myo1-GFP and MLD-GFP are shown below the localisation percentages. Scale bar denotes 5 μm.

In order to determine whether there is a discrete region of Myo1 necessary and sufficient for localisation, deletion constructs were created and transformed into wild type and *myoIΔ* strains. The number of rings at the bud neck as a percentage of the total number of bud necks was calculated for each construct in both wild-type and *Δmyo1 *strains and the results are shown in Fig 2, with fluorescent microscopy images of GFP labelled Myo1 and MLD shown below the localisation percentages. These results show that the minimum localisation domain (MLD) consists of residues 1044–1569. The coiled coil prediction plot shows that the MLD (indicated in Fig. 1) encompasses the region of low coiled coil forming probability, which includes 7 proline residues. Deletion of any additional parts of the MLD led to a lower percentage of localisation as compared to the full length MLD (Fig 2).

### The minimum localisation domain (MLD) of Myo1 forms an oligomer

**Figure 3:**
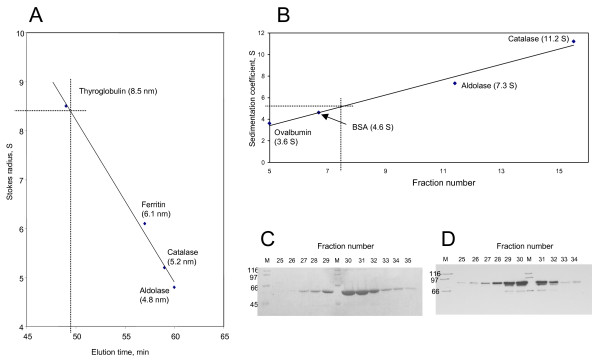
**Hydrodynamic analysis of Myo1 MLD A. **Superose 6 gel filtration calibration curve with positions of protein standards of known Stokes radii shown. The dotted lines show the position of the Myo1 MLD with a measured Stokes radius of 8.4 nm. **B. **Sucrose density gradient calibration curve with the positions of protein standards of known sedimentation coefficients shown. The dotted lines show the position of Myo1 MLD with a measured sedimentation coefficient of 5.2 S. **C. **Coomassie Blue stained SDS-PAGE gel showing Superose 6 FPLC fractions of bacterially expressed MLD (after GST tag removal). Fractions were TCA precipitated before loading on the gel. Numbers to the left of the gel show molecular weight of markers (M). **D. **Western blot of Superose 6 FPLC fractions of MLD-(myc)6 yeast extract using anti-myc antibodies. Fractions were TCA precipitated before loading on the gel. Horizontal black marks and numbers to the left of the marks show molecular weight of markers (M).

Since dimerisation and subsequently filament formation in many class II myosins is required for localisation we wanted to determine if the MLD forms oligomers. To this end, GST-MLD was purified after expression in *E. coli*. After removal of the GST tag, the MLD was applied to a Superose 6 gel filtration column. The MLD eluted at 49.4 +/- 0.4 minutes (n = 7) and the Stokes radius was obtained from a calibration curve of standards with known Stokes radii as shown in Fig. 3a; this resulted in a Stokes radius of 8.4 +/- 0.7 nm for the MLD (Fig. 3a and 3c). The void volume for the column was obtained using Dextran blue (2000 kDa, Stokes radius unknown), which gave an elution time of 32.2 minutes. Thyroglobulin, which has a molecular weight of 669 kDa and an elution time of 49.0 minutes, ran close to the MLD, showing that the calibration curve was well within the upper resolution limit of the column. Purified MLD was also applied to a preformed 6–20 % sucrose density gradient and was found in fraction 7.6 +/- 0.5 (n = 4). A sedimentation coefficient obtained from a calibration curve of known sedimentation coefficients, as shown in Fig. 3b, was 5.2 +/- 0.3 S (n = 4). Neither gel filtration nor sucrose density gradient centrifugation can be used in isolation to obtain the molecular weight of the MLD as the presumed elongated nature of the protein will result in a larger apparent Stokes radius and a smaller apparent sedimentation coefficient as compared with a globular protein. This makes it difficult to determine the true molecular weight and thus the oligomeric nature of the MLD. However, combining the Stokes radius and the sedimentation coefficient in Equation 1 (see Materials and Methods) allowed us to determine an experimental molecular weight of 177 kDa for the MLD. When this value is divided by the calculated molecular weight for a monomer (62 kDa), the oligomer is tentatively assigned to be a trimer (2.9 monomers).

We were concerned that the experimental values obtained were a product of expressing and purifying the MLD from *E. coli*. In order to be sure that our expressed protein was behaving in the same way as it would in yeast, the MLD with a C-terminal (myc)6 tag was transformed into yeast (RLY 1232). Yeast extracts were prepared by grinding frozen cells under liquid nitrogen using a pestle and mortar; the powder was resuspended in a small volume of buffer and centrifuged to remove cellular debris and unlysed cells. 250 μl of the supernatant was applied to the Superose 6 gel filtration column. Fractions were collected and western blots were carried out after trichloroacetic acid (TCA) precipitation to determine the elution position of MLD-(myc)6. As shown in Fig. 3c and 3d, MLD-(myc)6 eluted in the same fractions (48.2 +/- 0.4 minutes, n = 4) as the expressed bacterial protein (49.4 +/- 0.4 minutes) indicating that they are hydrodynamically similar and thus that the trimer value assigned to MLD was not an artefact of expression in *E. coli*.

### Removing the MLD hinge region affects the function of Myo1

We observed that deletion of the MLD from Myo1 resulted in complete loss of localisation (Fig. 2) but that deletion of the hinge region (residues 1162–1430) within the MLD only lowered the efficiency of localisation to the bud neck to 70 – 80 % of the full length molecule (Fig. 2) showing that the hinge region contributes to localisation but its loss does not abolish localisation. Since several other class II myosins contain kinks (where the coiled coil heptad repeat is interrupted) in their tail that are important for function, we wanted to examine the effect of removing the hinge region from Myo1 on CR contraction. A plasmid encoding Myo1Δhinge [Myo1(1–1161:1431–1928)-GFP-(myc)6] under the control of the *MYO1 *promoter was transformed into the heterozygous diploid *myo1Δ* strain (RLY621), and haploid strains straining expressing Myo1Δhinge as the sole Myo1 were obtained by sporulation and tetrad dissection.

**Figure 4:**
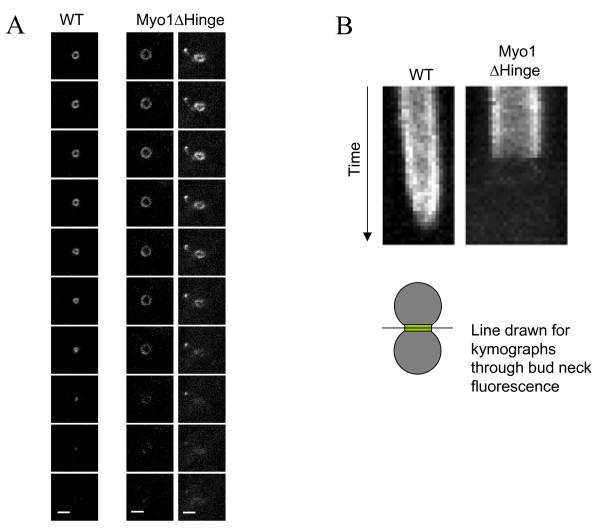
**Time-lapse imaging of the wild-type and Δhinge Myo1 ring contraction**. Typical examples of Myo1-GFP-(myc)6 (RLY1331) and Myo1Δhinge-GFP-(myc)6 (RLY2139) CR contraction as shown as a montage with images one minute apart **(A) **and as kymographs **(B)**. Each horizontal line of the kymograph represents a time point of the image series with images taken at 30 s intervals, derived from a line drawn across the GFP signal at the bud neck (shown in the schematic below B). These lines are then stacked next to each other in chronological order to generate the kymograph. In A, scale bars denote 5 μm.

We monitored contraction of the CR in wild-type and Myo1Δhinge strains. Cells were grown at the room temperature and arrested for 3 hrs with nocodazole. The cells were then released from arrest and contraction was monitored using time-lapse confocal microscopy with images taken every 30 s. Fig. 4 shows montages (A) and kymographs (B) of the wild-type and Myo1Δhinge ring as it goes through cytokinesis. Interestingly, Myo1Δhinge rings were unable to undergo contraction: the rings remained steadily at the bud neck until late in the cell cycle before gradually disappearing without reduction in diameter (n = 15). This was in contrast to the CR in wild-type cells, which contracted to a point before disappearing (n = 15).

### Fluorescence recovery after photobleaching (FRAP) studies of wild-type and mutant Myo1-GFP rings

Fluorescence recovery after photobleaching (FRAP) enables one to examine the dynamics of a fluorescently labelled molecular structure: fast recovery of the fluorescent signal indicates that there is a free pool of molecules that interchange rapidly with the molecules in the bleached area. We used FRAP to compare the *in vivo *dynamics of GFP-tagged full length Myo1, MLD and Myo1Δhinge [strains RLY1044, RLY1046 and RLY2139 (Table 2)]. Images were collected at 5 or 10 s intervals and analysed for fluorescence recovery. Table 1 shows the average percentage recovery and half time values. Myo1Δhinge appeared to be the most dynamic, recovering the greatest proportion of the bleached fluorescence and having the smallest recovery half time. This suggests that the contraction defect of the Myo1Δhinge could be due to a structural abnormality causing the mutant Myo1Δhinge molecules to be less well anchored within the CR. The MLD on the other hand appears to have a dynamic behaviour in between that of the full length molecule and Myo1Δhinge (Table 1) suggesting that the MLD is sufficient for stable association with the CR, but that other regions of Myo1 further stabilise the association.

## Discussion

In the experiments described above, we have characterized the minimum domain required for targeting myosin-II to the site of cell division in budding yeast. We show that this domain can oligomerise and form what appears to be trimers in solution and exhibits dynamics intermediate to those of the full length Myo1 molecule and Myo1 missing the region of low coiled coil forming probability (Myo1Δhinge).

How feasible is it that Myo1 might form a trimer? Coiled coils are a ubiquitous protein motif with the ability to form many different kinds of structures depending on the knobs-into-holes packing of the residues in the coiled coil and how this is disrupted by the distribution of breaks and stutters within the heptad repeat sequence [16]. The formation of triple stranded coiled coils is not unusual [16]; for example the influenza hemagglutinin protein [17] and the bacteriophage T4 whisker proteins [18] form parallel triple stranded coiled coils while the BAR domain of proteins such as amphiphysin consists of an antiparallel triple stranded coiled coil that dimerises [19]. Additionally trimers may be observed experimentally even when they have a dimeric coiled coil prediction [20,21]. Kammerer et al [21] provide evidence for a 5 residue trimerisation motif (R- [ILVM]-X-X- [ILV]-E) in short coiled coils. Interestingly two such motifs are present in the Myo1 tail (RVKSLE and RLSAVE at residues 1377–1382 and 1575–1580 respectively), one at the C-terminal end of the hinge region and one just beyond the C-terminal end of the MLD. Furthermore, many of the **a **and **d **residues of the heptad repeat in the Myo1 tail as predicted by either COILS [22,23] or PAIRCOIL [15] are asparginine, threonine and serine residues which can be prevalent in trimeric coiled coil structures [21].

In order to confirm the trimeric nature of the MLD, electron microscopy and analytical ultra centrifugation experiments were performed. Preliminary electron microscopy (EM) negative stain studies (in collaboration with D. Hanein and L. Hazelwood – results not shown) of purified MLD show a variety of oligomers which vary in length. Analytical ultra centrifugation experiments showed only protein aggregation indicating an inherent stickiness in the protein. Interestingly the elution peaks in gel filtration and sucrose density gradient were well defined and narrow (Figure 3b and 3c) indicating a single species of oligomer and also suggesting that the conditions necessary for EM or analytical centrifugation lead to protein instability. We speculate that the coiled coil domains present in the MLD are not long enough to cause stable association but that the MLD may be a minimal oligomerisation unit and possibly also a filament nucleation unit. That the MLD represents a normal assembly competent form is supported by the FRAP data which shows that the structure formed by the MLD was dynamically similar to that formed by full length Myo1 (Table 1). However further understanding of the nature and minumum oligomerisation unit (dimer or trimer) requires detailed structural and biochemical analysis of the native Myo1. If the trimeric nature suggested by our experiments of the MLD are indeed a property of the full length molecule, this would suggest a novel mode of contraction for the budding yeast CR as a Myo1 trimer would have 3 motor domains as compared with the two motor domains present in a more conventional dimeric structure.

As well as containing a region in the tail necessary for filament formation, all non-muscle myosins also contain tail sequences that disrupt the coiled coil formation and cause the tail to kink or bend sharply [7]. In higher eukaryotes bending at these sites is regulated by light chain phosphorylation, whereas in *D. discoideum *bending is regulated by heavy chain phosphorylation. Phosphorylation causes the tail to bend into a folded conformation that prevents filament formation [7]. We have shown that the region of low coiled coil forming probability in Myo1, termed the hinge, is important for CR contraction. The FRAP data also show that fluorescence recovery of Myo1Δhinge is faster than that of the wild-type molecule. It is interesting that the MLD, which possesses the hinge region, has a slower exchange half time than Myo1Δhinge, suggesting that the hinge has a role in anchoring Myo1 in the CR. Taken together these data suggest that removal of the hinge prevents proper CR assembly, resulting in reduced structural stability. The inability of the hingeless CR to contract could either be due to a lack of force generation or due to rapid disassembly when under force. Interestingly, Lord et al [24] have recently shown that the motor domain of Myo1 is not necessary for contraction to occur at the bud neck during cytokinesis in budding yeast, though the rate of contraction is slower than wild type. This suggests that there is an additional force generating element in the budding yeast CR and that the Myo1 tail may serve to guide this force. The hinge region may play a direct role in linking the two force generating elements. The additional force generating element acting alone in the absence of Myo1 or in the presence of a defective Myo1 could come from Chs2, the enzyme that synthesizes and deposits chitin during cytokinesis and this synthetic energy could be used to drive membrane closure [1,25,26]. Normally this force would be coupled with the force generated by Myo1 and we speculate that the tail of Myo1 may interact directly with Chs2. Future studies will examine the localisation of other bud neck components in the presence of the MLD or the hingeless Myo1 to see if their localisation is perturbed. This will further our understanding of the cytokinetic defects caused by the loss of hinge in Myo1 and of how it interacts with other bud neck components.

Finally, how does the MLD compare with the localisation determinants of other class II myosins? In fission yeast Myo2, the region required for localization is different: it was shown that a small C-terminal domain is necessary and sufficient for localisation to the contractile ring [8,13]. Phosphorylation plays a regulatory role in Myo2 localisation, though there is disagreement over the exact residues involved. There is also evidence that the very C-terminus of budding yeast Myo1 is phosphorylated [27], but the precise residues and function of this phosphorylation are not known. Our results showing localisation in a Myo1 construct lacking the C-terminus (Myo1 1–1908, Fig. 2) suggest that this phosphorylation would not be required for Myo1 localisation.

It remains open whether myosin-II recruitment is mediated by a specific interaction, with certain components of the cell division site, or by an interaction that requires certain structural features of the myosin-II complex assembly. Regardless, the MLD can be used as bait for the identification of the binding partners responsible for the recruitment of myosin-II to the bud neck. However, given the potential requirement for structural determinants and the fact that direct recruiting factors for myosin-II have yet to be found, we speculate that recruitment of myosin-II may be mediated by multiple low affinity interactions that rely on structural assembly of the CR and may not be detectable by conventional solution-based assays for protein interactions.

## Conclusion

Our results show that a small defined region in tail of budding yeast myosin II, the MLD, is necessary and sufficient for localisation. We show that when expressed in bacteria or in yeast, this domain behaves like a trimer rather than like the more traditional class II myosin dimer, raising the possibility that if the trimeric nature holds for the full length Myo1, the structure of the CR in budding yeast might be different than were it composed in part of a dimeric myosin II. The similarity between the dynamics of the MLD and full length budding yeast myosin II *in vivo *as explored through our FRAP studies lends confidence that the MLD assembly *in vivo *is structurally similar to that of full length Myo1. We also show that the MLD encompasses a region of low coiled coil forming probability (the hinge) which, when removed, does not abolish myosin II localisation to the bud neck, the site of cell division, but does prevent proper contraction of the CR during cytokinesis. We propose that this hinge region may interact directly with other components of the CR and that its loss leads to a more dynamic, less anchored molecule as shown by the faster fluorescent turnover seen in our FRAP studies in comparison with the MLD and full length protein.

## Methods

### Media and genetic manipulations

Yeast cell culture and genetic techniques were carried out by methods described in [28]. Yeast extract, peptone, dextrose (YPD) contained 2% glucose, 1% yeast extract and 2% Bactopeptone (Difco Laboratories, Detroit, MI). Synthetic complete (SC) media was prepared by the method described [29].

### Plasmid construction

The plasmid expressing Myo1-GFP-(myc)6 (pNT50) was generated by excising *MYO1 *from a previously-described plasmid encoding Myo1-GFP (pLP8 [30]) with *Pst*I and *Bam*HI, and ligating it into *Pst*I-*Bam*HI-digested pRL304, a pRS315-based vector for C-terminal tagging with a GFP-(myc)6 fusion. To generate Myo1-GFP-(myc)6 in a pRS316 (URA3)-based vector, the 7 kb fragment from *Sal*I-*Not*I digested pNT50 was ligated to *Sal*I-*Not*I digested pRS316, creating pNT113. To generate the plasmid that expresses the C-terminal 884 amino acids of Myo1p under the control of the *MYO1 *promoter with a C-terminal GFP tag (pNT13), the sequence encoding the promoter region and the first 1043 amino acids was excised from pLP8, using *Pst*I and *Bgl*II, and replaced with just the promoter region (generated by PCR and digested with *Pst*I and *Bgl*II). The GFP tag of pNT13 was replaced with a GFP-(myc)6 fusion by ligating the 1 kb fragment generated by *Bam*HI-*Not*I digest of pNT50 to *Bam*HI-*Not*I digested pNT13, creating pNT76. To create the plasmid expressing Myo1(1044–1569), pNT13 was double digested with *Bgl*II and *Hae*III yielding a 1.6 kb fragment encoding Myo1(1044–1569). pNT13 was digested with *Bam*HI, blunted and digested with *Bgl*II, to yield a 7 kb fragment encoding the vector backbone, *MYO1 *promoter, and GFP. Ligation of both fragments produced an in-frame fusion of Myo1(1044–1569) and GFP (pNT43), which was then excised using *Pst*I and *Bam*HI, and ligated into *Pst*I-*Bam*HI-digested pRL304 to create Myo1(1044–1569)-GFP-(myc)6 (pNT49). The sequence encoding Myo1(1529–1928) was generated by PCR from yeast genomic DNA and digested with *Bgl*II and *Bam*HI (restriction sites contained within the forward and reverse primers, respectively), and ligated to the 6.9 kb fragment produced by *Bgl*II-*Bam*HI double digest of pNT13, to generate Myo1(1529–1928)-GFP (pNT40). To create the plasmid expressing Myo1(1–1043), pNT50 was double-digested with *Bgl*II and *Bam*HI, blunted and self-ligated yielding an in-frame fusion of Myo1(1–1043) and GFP-(myc)6 (pNT51). The sequence encoding Myo1(1044–1253) was generated by digesting pNT43 with *Xba*I, blunting, and digesting with *Pst*I, yielding a 0.9 kb fragment. This fragment was ligated to pNT49 that had been digested with *Bam*HI, blunted, and digested with *Pst*I, to create Myo1(1044–1253)-GFP-(myc)6 (pNT62). pNT43 was digested with *Xba*I, blunted and digested with *Bam*HI to produce a 0.9 kb fragment encoding Myo1(1261–1569). This was ligated to pNT49 that had been digested with *Bgl*II, blunted, and digested with *Bam*HI, to create Myo1(1261–1569)-GFP-(myc)6 (pNT63). A construct expressing Myo1 lacking the C-terminal 26 amino acids was generated by utilizing an existing *Eco*RI site, as described previously [27]. Briefly, *MYO1*, cloned *Pst*I-*Bam*HI in the Bluescript vector (pLP9), was digested with *Eco*RI to yield a 6 kb fragment encoding Myo1(1–1902). This fragment was ligated to *Eco*RI-digested pRL164, a pRS313-based vector for C-terminal GFP tagging, to generate pRL221. This plasmid was double-digested with *Bgl*II and *Bam*HI to generate a fragment encoding Myo1(1044–1902), and this was ligated to *Bgl*II-*Bam*HI digested pNT50 to create Myo1(1–1902)-GFP-(myc)6 (pNT66). The sequence encoding Myo1(1044–1161) was generated by PCR from yeast genomic DNA and digested with *Bgl*II and *Bam*HI (restriction sites contained within the forward and reverse primers, respectively) and ligated to the 6.9 kb fragment produced by *Bgl*II-*Bam*HI double digest of pNT13 to generate Myo1(1044–1163)-GFP (pNT69). The GFP tag of pNT69 was replaced with a GFP-(myc)6 fusion by ligating the 1 kb fragment generated by *Bam*HI-*Not*I digest of pRL304 to *Bam*HI-*Not*I digested pNT69, creating pNT71. The sequences encoding Myo1(1160–1928), Myo1(1044–1430), Myo1(1044–1387) and Myo1(1044–1314) were generated by PCR from yeast genomic DNA, digested with *Bgl*II and *Bam*HI (restriction sites contained within the forward and reverse primers, respectively) and ligated to the 7.3 kb fragment produced by *Bgl*II-*Bam*HI double digest of pNT76, to generate pNT94, pNT110, pNT111 and pNT112, respectively. Similarly, the sequence encoding Myo1(864–1161) was generated by PCR from yeast genomic DNA, digested with *Bam*HI (restriction site contained within the forward and reverse primers) and ligated to the 7.3 kb fragment produced by *Bgl*II-*Bam*HI double digest of pNT76, to generate Myo1(864–1161)-GFP-(myc)6 (pNT96). To create a deletion of the non-helical sequences within Myo1-tail, pNT121 was constructed. Briefly, the sequence encoding Myo1(1431–1928) was generated by PCR from yeast genomic DNA, digested with *Bam*HI (restriction site contained within the forward and reverse primers) and ligated to *Bam*HI digested pNT71, to generate Myo1(1044–1160:1431–1928)-GFP-(myc)6. Similarly, the sequence encoding Myo1(1570–1928) was generated by PCR from yeast genomic DNA, digested with *Bgl*II and *Bam*HI (restriction sites contained within the forward and reverse primers, respectively) and ligated to *Bgl*II-*Bam*HI digested pNT50, to generate Myo1(1–1044:1670–1928)-GFP-(myc)6 (pNT120). To create a deletion of the non-helical region of the MLD in the full length Myo1, pIL11 was constructed. Briefly pNT121 was cut with *Pst*I/*Bgl*II to yield a ~7000 bp fragment encoding Myo1(1044–1161:1439–1928). This fragment was ligated with a ~3300 bp fragment encoding Myo1 promoter -Myo1(1–1043) cut with *Pst*I/*Bgl*II from pLP8. To create a plasmid (pIL10) encoding N-terminally tagged GST-MLD, a PCR product encoding Myo1(1044–1559) was cut with *Bam*HI/*EcoR*I was ligated into pGEX6P1 that contains a precision protease cleavage site between the GST and MLD reading frames. To create an MLD construct C-terminally tagged with (myc)6 for expression in yeast, a fragment consisting of Myo1 promoter-Myo1(1044–1569) was cut from pNT49 using *Pst*I and *Bam*HI and ligated into pRL72 cut *Pst*I/*Bam*HI resulting in pIL13.

### Quantification of Myo1-GFP and deletion construct bud neck localization

Exponentially growing cultures of cells expressing either Myo1-GFP or a deletion construct were assessed for bud neck localization using an Eclipse E800 microscope with a 100/1.40 oil differential-interference contrast objective (Nikon, Melville, NY). At least 100–200 live cells were analyzed, and data shown are averages from at least 3 independent experiments. Images were collected with a 0.5 s exposure to fluorescent light filtered through an EXHQ450/50 DM480 LP/BA465LP GFP filter set (Chroma, Brattleboro, VT) with a cooled RTE/CCD 782Y Interline camera (Princeton Instruments) using MetaMorph (Universal Imaging Corp., West Chester, PA). RLY2127 and 2128 fluorescence was weak and could not be collected using the method described above. Instead 20 z sections at 0.5 μm intervals were collected and deconvolved as described below for the CR movies. RLY1044 and RLY1046 were also measured in this way to ensure that counts were comparable with those obtained by the first method (Results for RLY1044: 79 %. Results for RLY1046: 75 %).

### Fluorescence recovery after photobleaching (FRAP)

FRAP experiments were carried out using a 435 nm nitrogen pulsed Micropoint laser (Photonic Instruments Inc., St. Charles IL). Galvanometer based steering optics controlled with MetaMorph software were used to position the laser beam, which was focused into a diffraction-limited 0.7 μm spot using a Nikon 100× 1.4 N.A. objective lens. Images were acquired every 5 or 10 s with a Yokogawa spinning disc confocal microscope mounted on a Nikon TE2000U stand and a Hamamatsu ORCA ER cooled CCD. All measurements were done using MetaMorph and all data analysis was done using Excel (Microsoft). To analyse the data, the signal from the FRAPed bud neck and a control bud neck (to check for bleaching) were collected. Regression analysis to determine the half time to maximum was done using a one-phase exponential association function (Y = bottom + (top-bottom)·(1-exp [-k·x]), where k is the rate constant and *t*1/2 is 0.69/k in Prism (version 4.00; GraphPad).

### Time-lapse imaging of CR contraction

For assaying CR contraction, cells were arrested in metaphase with nocodazole for 3 hrs at room temperature and then released from the arrest. The released cells were mounted on gelatine pads. Three dimensional (3D) images and movies of RLY1331 and RLY2139 were collected with the spinning disc confocal microscope described above, imaging 12 sections in the z-plane with a spacing of 0.5 μm, with exposures of 1 s. Each stack of z-series images was deconvolved and rendered in to 3D images and movies using MetaMorph (Universal Imaging Corp., West Chester, PA).

### Protein purification

Myo1 minimum localisation domain (MLD) tagged N-terminally with GST (plasmid pIL10) was transformed into BL21 cells and grown in 1.5 L cultures at 37°C until an OD_600 _of 1–1.2. The temperature was reduced to 24°C and IPTG was added to a final concentration of 50 μM before growing the cultures for a further 4 hrs. The cells were harvested and stored as pellets at -20°C. Pellets were lysed in 25 % sucrose, 50 mM Tris HCl, pH 7.5, 500 mM NaCl, 1 Roche inhibitor tablet per 50 ml solution, 2 mM DTT. The lysate was centrifuged at 35,000 rpm for 30 minutes and the supernatant added to 800 μl Glutathione Sepharose 4B (Amersham Biosciences) for 3 hrs at 4°C with constant agitation. The resin was washed with 50 ml buffer A+ Triton (500 mM NaCl, 50 mM Tris HCl pH 7.5, 1 mM DTT, 1 % Triton X-100) followed by 40 ml buffer A (500 mM NaCl, 50 mM Tris HCl pH 7.5, 1 mM DTT). The resin was resuspended in 300–500 μl buffer A and 30 μl of GST tagged precision protease at 0.6 mg/ml was added. Digestion was carried out overnight at 4°C and the supernatant was removed after low speed centrifugation to pellet the resin.

### Gel filtration analysis

A Superose 6 column (BioRad) was equilibrated in buffer A. 250 μl of Glutathione sepharose purified MLD with the GST tag removed was added and 0.5 ml fractions were collected. Alternatively, 250 μl RLY2132 yeast grinding extract (see below) was run over the column. For SDS-PAGE analysis, 10 % TCA was added to each fraction, incubated on ice for 30 minutes and centrifuged for 15 minutes. The pellets were resuspended in 30 μl SDS-loading buffer and 5 μl 1 M non-pH'd Tris base. MLD-(myc)6 from yeast extract fractions run on the column was detected by Western blotting using the anti-myc monoclonal antibodies, 9E-10. The column was calibrated with globular proteins of known Stokes radii: Thyroglobulin – 669 kDa, 8.5 nm, Ferritin – 440 kDa, 6.1 nm, Catalase – 232 kDa, 5.2 nm, Aldolase 158 kDa, 4.8 nm. Dextran Blue with a molecular weight of approximately 2000 kDa was used to establish the void volume of the column.

### Sucrose density centrifugation gradients

5 ml 6–20 % sucrose gradients were prepared in ultra clear centrifuge tubes (Beckman) for the SWTi55 rotor (Beckman). The gradient was calibrated using protein standards of known sedimentation coefficients: catalase – 232 kDa, 11.2 S, aldolase – 158 kDa, 7.3 S, BSA – 66 kDa, 4.6 S, ovalbumin – 43 kDa, 3.6 S. The top 400 μl of the gradient were removed and replaced with 300 μl Glutathione sepharose 4B purified MLD, 5 μg of aldolase as an internal control, sucrose to a final concentration of 6 % and water to 400 μl. The gradient was centrifuged for 19 hrs at 40 k rpm at 4°C. At the end of the run, the gradient was fractionated from the top into 250 μl fractions. These were TCA precipitated as described above.

### Calculation of native molecular weight

The native molecular weight of the expressed MLD was calculated using its Stokes radius measured by gel filtration and its sedimentation coefficient determined by sucrose density gradient centrifugation using the following equation 1 described in [31].

M.W=Sω20N.6π.n.Rs1−ν¯ρ
 MathType@MTEF@5@5@+=feaafiart1ev1aaatCvAUfKttLearuWrP9MDH5MBPbIqV92AaeXatLxBI9gBaebbnrfifHhDYfgasaacH8akY=wiFfYdH8Gipec8Eeeu0xXdbba9frFj0=OqFfea0dXdd9vqai=hGuQ8kuc9pgc9s8qqaq=dirpe0xb9q8qiLsFr0=vr0=vr0dc8meaabaqaciaacaGaaeqabaqabeGadaaakeaacqqGnbqtcqqGUaGlcqqGxbWvcqGH9aqpdaWcaaqaaiabdofatHGaciab=L8a3naaBaaaleaacqaIYaGmcqaIWaamaeqaaGqacOGae4Nta4KaeeOla4IaeGOnayJae8hWdaNaeiOla4IaeeOBa4MaeeOla4IaeeOuai1aaSbaaSqaaiabbohaZbqabaaakeaacqaIXaqmcqGHsislcuWF9oGBgaqeaiab=f8aYbaaaaa@462F@

where *Sω*_20 _is the sedimentation coefficient, R_*s *_is the Stokes radius, Avogadro's number, *N *is 6.02 × 10^23^; viscosity coefficient, n is 1×10^-2 ^g s^-1 ^cm^-1^; solution density, ρ is 1 g cm^-3 ^and partial specific volume, ν¯
 MathType@MTEF@5@5@+=feaafiart1ev1aaatCvAUfKttLearuWrP9MDH5MBPbIqV92AaeXatLxBI9gBaebbnrfifHhDYfgasaacH8akY=wiFfYdH8Gipec8Eeeu0xXdbba9frFj0=OqFfea0dXdd9vqai=hGuQ8kuc9pgc9s8qqaq=dirpe0xb9q8qiLsFr0=vr0=vr0dc8meaabaqaciaacaGaaeqabaqabeGadaaakeaaiiGacuWF9oGBgaqeaaaa@2E83@ is 0.71 cm^3 ^g^-1 ^(assumed to be that of an average soluble protein).

### Yeast extracts

To prepare the yeast extracts used for gel filtration analysis, small pellets were extruded through a syringe into N_2 _(l) creating cylindrical fragments that could then be ground by pestle and mortar in the presence of N_2 _(l). The powdered yeast was thawed and resuspended in buffer A. Protease inhibitors (2 μg/ml Antipain, 2 μg/ml Leupeptin, 2 μg/ml Aprotinin, 2 μg/ml Pepstatin, 2 μg/ml Chymostatin) and 1 mM PMSF were added to both buffers. Lysis was checked using light microscopy. Thawed extracts were centrifuged at 50 K rpm in a Sorvall RP120AT rotor for 30 minutes at 4°C.

## Authors' contributions

NT created the plasmids and strains and performed the experiments to assess bud neck localization of the different Myo1 constructs, with the exception of pIL11 and RLY2127 and RLY 2128. Other strains containing pIL11 were created by IMBL. IMBL performed all other experiments and drafted the manuscript. RL conceived of the study, participated in its design and coordination. IMBL, NT and RL revised the manuscript. All authors read and approved the final manuscript.

## Figures and Tables

**Table 1:** FRAP total fluorescence recovery and half times

Strain	Construct in Δmyo1 background	Percentage recovery (SD)	Half time (SD), s	Number of cells
RLY1044	Myo1-GFP-6myc	27 (11)	79 (44)	14
RLY1046	MLD-GFP-6myc	40 (14)	50 (38)	11
RLY2139	Myo1Δhinge-GFP-6myc	50 (18)	28 (18)	12

**Table 2:** Strains

Name		Genotype	Background	Source
RLY261		*MATa ura3-1 his3-11,15 leu2-3,112 trp1 ade2-1 bar1Δ*	W303a	Elion Lab
RLY621		*MATa/α ura3/ura3 his3/his3 leu2/leu2 trp1/trp1 myo1Δ::TRP1/MYO1*	S288c	Li Lab
RLY622		*MATα ura3 his3 leu2 trp1 myo1Δ::TRP1*	S288c	Li Lab
RLY751		*MATa ura3-1 his3-11,15 leu2-3,112 trp1-1 ade2-1 bar1Δ pMYO1(1044–1928)-GFP:LEU2 (pNT13)*	W303a	This work
RLY1034		*MATa ura3-1 his3-11,15 leu2-3,112 trp1-1 ade2-1 bar1Δ pMYO1(1044–1569)-GFP-6myc:LEU2 (pNT49)*	W303a	This work
RLY1035		*MATa ura3-1 his3-11,15 leu2-3,112 trp1-1 ade2-1 bar1Δ pMYO1-GFP-6myc:LEU2 (pNT50)*	W303a	This work
RLY1040		*MATa/α ura3-1/ura3-1 his3-11,15/his3-11,15 leu2-3,112/leu2-3,112 trp1-1/trp1-1 ade2-1/ade2-1 bar1Δ/bar1Δ myo1Δ::TRP/MYO1*	W303a	This work
RLY1044		*MATα ura3 his3 leu2 trp1 myo1Δ::TRP1 pMYO1-GFP-6myc:LEU2 (pNT50)*	S288c	This work
RLY1046		*MATα ura3 his3 leu2 trp1 myo1Δ::TRP1 pMYO1(1044–1569)-GFP-6myc:LEU2 (pNT49)*	S288c	This work
RLY1051		*MATa ura3-1 his3-11,15 leu2-3,112 trp1-1 ade2-1 bar1 Δ pMYO1(1–1043)-GFP-6myc:LEU2 (pNT51)*	W303a	This work
RLY1073		*MATa ura3-1 his3-11,15 leu2-3,112 trp1-1 ade2-1 bar1Δ pMYO1(1044–1253)-GFP-6myc:LEU2 (pNT62)*	W303a	This work
RLY1074		*MATa ura3-1 his3-11,15 leu2-3,112 trp1-1 ade2-1 bar1Δ pMYO1(1261–1569)-GFP-6myc:LEU2 (pNT63)*	W303a	This work
RLY1096		*MATa/α ura3-1/ura3-1 his3-11,15/his3-11,15 leu2-3,112/leu2-3,112 trp1-1/trp1-1 ade2-1/ade2-1 bar1Δ/bar1Δ myo1Δ::TRP/MYO1 pMYO1(1-1902)-GFP-6myc:LEU2 (pNT66)*	W303a	This work
RLY1101		*MATa ura3-1 his3-11,15 leu2-3,112 trp1-1 ade2-1 bar1Δ pMYO1(1–1902)-GFP-6myc:LEU2 (pNT66)*	W303a	This work
RLY1103		*MATa ura3-1 his3-11,15 leu2-3,112 trp1-1 ade2-1 bar1Δ myo1Δ::TRP pMYO1(1–1902)-GFP-6myc:LEU2 (pNT66)*	W303a	This work
RLY1119		*MATa ura3-1 his3-11,15 leu2-3,112 trp1-1 ade2-1 bar1Δ pMYO1(1044–1161)-GFP-6myc:LEU2 (pNT71)*	W303a	This work
RLY1212		*MATα ura3 his3 leu2 trp1 myo1Δ::TRP1 pMYO1(1044–1928)-GFP-6myc:LEU2 (pNT76)*	S288c	This work
RLY1288		*MATa ura3-1 his3-11,15 leu2-3,112 trp1-1 ade2-1 bar1Δ pMYO1(1161–1928)-GFP-6myc:LEU2 (pNT94)*	W303a	This work
RLY1295		*MATa ura3-1 his3-11,15 leu2-3,112 trp1-1 ade2-1 bar1Δ pMYO1(864–1161)-GFP-6myc:LEU2 (pNT96)*	W303a	This work
RLY1295		*MATa ura3-1 his3-11,15 leu2-3,112 trp1-1 ade2-1 bar1Δ pMYO1(864–1161)-GFP-6myc:LEU2 (pNT96)*	W303a	This work
RLY1297		*MATa ura3-1 his3-11,15 leu2-3,112 trp1-1 ade2-1 bar1Δ pMYO1(1529–1928)-GFP:LEU2 (pNT40)*	W303a	This work
RLY1298		*MATα ura3 his3 leu2 trp1 myo1Δ::TRP1 pMYO1(1529–1928)-GFP:LEU2 (pNT40)*	S288c	This work
RLY1307		*MATa ura3-1 his3-11,15 leu2-3,112 trp1-1 ade2-1 bar1Δ pMYO1(1044–1430)-GFP-6myc:LEU2 (pNT110)*	W303a	This work
RLY1308		*MATa ura3-1 his3-11,15 leu2-3,112 trp1-1 ade2-1 bar1Δ pMYO1(1044–1387)-GFP-6myc:LEU2 (pNT111)*	W303a	This work
RLY1309		*MATa ura3-1 his3-11,15 leu2-3,112 trp1-1 ade2-1 bar1Δ pMYO1(1044–1314)-GFP-6myc:LEU2 (pNT112)*	W303a	This work
RLY1310		*MATα ura3 his3 leu2 trp1 myo1Δ::TRP1 pMYO1(1044–1430)-GFP-6myc:LEU2 (pNT110)*	S288c	This work
RLY1311		*MATα ura3 his3 leu2 trp1 myo1Δ::TRP1 pMYO1(1044–1387)-GFP-6myc:LEU2 (pNT111)*	S288c	This work
RLY1312		*MATα ura3 his3 leu2 trp1 myo1Δ::TRP1 pMYO1(1044–1314)-GFP-6myc:LEU2 (pNT112*	S288c	This work
RLY1330		*MATa/α ura3-1/ura3-1 his3-11,15/his3-11,15 leu2-3,112/leu2-3,112 trp1-1/trp1-1 ade2-1/ade2-1 bar1Δ myo1Δ::KAN/MYO1 pMYO1-GFP-6myc:LEU2 (pNT113)*	W303a	This work
RLY1331		*MA/α ura3-1 his3-11 leu2-3,112 trp1-1 ade2-1 bar1Δ myo1Δ::KAN pMYO1-GFP-6myc:LEU2 (pNT113)*	W303a	This work
RLY1577		*MATa ura3-1 his3-11,15 leu2-3,112 trp1-1 ade2-1 bar1Δ pMYO1(1044–1160:1431–1928)-GFP-6myc:LEU2 (pNT121)*	W303a	This work
RLY1578		*MATα ura3 his3 leu2 trp1 myo1Δ::TRP1 pMYO1(1044–1160:1431–1928)-GFP-6myc:LEU2 (pNT121)*	S288c	This work
RLY1591		*MATα ura3 his3 leu2 trp1 myo1Δ::TRP1 pMYO1(1161–1928)-GFP-6myc:LEU2 (pNT94)*	S288c	This work
RLY1598		*MATa ura3-1 his3-11,15 leu2-3,112 trp1-1 ade2-1 bar1Δ myo1Δ::TRP pMYO1-GFP-6myc:URA3 (pNT113)*	W303a	This work
RLY1653		*MATa ura3-1 his3-11,15 leu2-3,112 trp1-1 ade2-1 bar1Δ myo1Δ::TRP pMYO1-GFP-6myc:URA3 (pNT113) p:LEU2 (pRL304)*	W303a	This work
RLY1655		*MATa ura3-1 his3-11,15 leu2-3,112 trp1-1 ade2-1 bar1Δ myo1Δ::TRP pMYO1-GFP-6myc:URA3 (pNT113) g pMYO1-GFP-6myc:LEU2 (pNT50)*	W303a	This work
RLY1657		*MATa ura3-1 his3-11,15 leu2-3,112 trp1-1 ade2-1 bar1Δ myo1Δ::KAN pMYO1-GFP-6myc:URA3 (pNT113) pMYO1(1–1045:1570–1928)-GFP-6myc:LEU2 (pNT120)*	W303a	This work
RLY1659		*MATa ura3-1 his3-11,15 leu2-3,112 trp1-1 ade2-1 bar1Δ pMYO1(1–1045:1570–1928)-GFP-6myc:LEU2 (pNT120)*	W303a	This work
RLY1660		*MATα ura3 his3 leu2 trp1 myo1Δ::TRP1g pMYO1(1–1045:1570–1928)-GFP-6myc:LEU2 (pNT120)*	S288c	This work
RLY2127		*MATα ura3 his3 leu2 trp1 myo1Δ::TRP, pMYO1(1–1160:1431–1928)-GFP-6myc:LEU (pIL11)*	S288c	This work
RLY2128		*MATa ura3-1 his3-11,15 leu2-3,112 trp1-1 ade2-1 bar1Δ pMYO1(1–1160:1431–1928-GFP-6myc):LEU (pIL11)*	W303a	This work
RLY2132		*MATα ura3 his3 leu2 trp1 MLD-6myc:LEU (pIL13)*	S288c	This work
RLY2139		*MATa ura3 his3 leu2 trp1myo1Δ::TRP1 pMYO1(1–1160:1431–1928) -GFP-6myc:LEU (pIL11)*	S288c	This work
